# An Interactive Narrative Intervention to Increase Adolescents’ Knowledge and Self-Efficacy to Reduce Alcohol Use: Pre-Post Study

**DOI:** 10.2196/83029

**Published:** 2026-08-10

**Authors:** Carlos Penilla, Mark S Berna, Marianne Pugatch, Vikram Kumaran, Sean Hennigan, Kathleen P Tebb, Sara Buckelew, Anoushka Sinha, Jonathan P Rowe, Alison Giovanelli, James C Lester, Courtney Barron, Leslie Einhorn, Elizabeth M Ozer

**Affiliations:** 1 Department of Pediatrics, University of California San Francisco, 550 16th Street, #4241, San Francisco, CA, 94158, United States, 1 415-502-2606; 2 Department of Psychiatry and Behavioral Medicine, Northwestern University Feinburg School of Medicine, Chicago, IL, United States; 3 Potocsnak Family Division of Adolescent and Young Adult Medicine, Department of Pediatrics and Pritzker Department of Psychiatry and Behavioral Health, Ann & Robert H. Lurie Children's Hospital of Chicago, IL, United States; 4 Department of Computer Science, North Carolina State University, Raleigh, NC, United States; 5 Applied Research Associates, Raleigh, NC, United States; 6 Children’s After School Arts, San Francisco, CA, United States; 7 Office of Opportunity and Outreach, University of California, , San Francisco, CA, United States

**Keywords:** adolescents, alcohol use, interactive narrative, game technology, clinic-based intervention

## Abstract

**Background:**

Accidents and injuries are the leading causes of preventable death among adolescents and are often related to substance use. About 60% of US high school students have tried alcohol and 22% report current alcohol use. Preventing and reducing adolescent alcohol use would contribute to substantial health benefits and prevent major health morbidity and mortality. Advances in interactive narrative learning technologies hold promise for designing games for health that effectively deliver age-appropriate and personalized behavior change interventions. The Interactive Narrative System for Patient-Individualized Reflective Exploration (INSPIRE) is designed to serve as an extension to clinical preventive care, engaging adolescents in a theoretically grounded alcohol prevention intervention by leveraging the dual mechanisms of interactive narrative and 3D game technologies.

**Objective:**

This pre-post study aims to examine the impact of INSPIRE on adolescents’ self-efficacy to avoid risky alcohol-related behavior and knowledge about alcohol risk.

**Methods:**

A total of 44 adolescents in high school (aged 14‐16 years; mean 15.16, SD 0.95; n=22, 50% female) were recruited using convenience sampling from an after-school program in the San Francisco Bay Area. The largest proportion of participants identified as Hispanic or Latine (n=15, 34%), followed by White, Asian, and multiple racial or ethnic backgrounds. Participants completed two 20-minute web-based interactive narrative episodes. We compared pretest and posttest data to examine changes in adolescents’ self-efficacy and knowledge using a combination of questionnaire and computer interaction trace log data. Self-efficacy was measured using a 24-item scale (*α*=.95; 0‐10 rating). Knowledge was assessed using 10 multiple-choice items derived from in-game content. Pre-post changes were analyzed using Wilcoxon signed-rank tests (*α*=.05), with rank-biserial correlation effect sizes and 95% CIs.

**Results:**

Approximately 25% (n=11, 95% CI 14.6%-39.4%) of study participants reported having consumed alcohol at least once, and 23% (n=10, 95% CI 12.8%-37.0%) reported alcohol use within the past year. Self-efficacy scores significantly increased from 7.97 (SD 2.24) at pretest to 8.72 (SD 1.58) at posttest, with a mean difference of 0.75 (95% CI 0.59‐0.91; *P*<.001; *r*=0.89). Knowledge scores also significantly increased from 5.09 correct (median 5.0, IQR 4.0-6.0) at pre‐test to 6.11 correct (median 7.0, IQR 5.0-8.0) at posttest, with a mean increase of 1.02 (95% CI 0.31‐1.74; Wilcoxon signed-rank test, *P*<.001; *r*=0.57). Reflection tool clustering revealed 4 behavioral strategy endorsement profiles.

**Conclusions:**

As a first of its kind interactive narrative intervention, INSPIRE offers an innovative theoretically grounded model for supporting adolescent health behavior change. This study enhances our understanding of how to use innovative learning technologies to reduce risky alcohol use. Extending prior research in the field through using personalized narrative adaptations, this study indicates that through reinforcing goals and decisions to avoid risky behavior, adolescents can enhance their self-efficacy beliefs to avoid risky alcohol use and increase their knowledge about alcohol risk. Implications of these outcomes include the potential to facilitate the generalization of preventive behaviors to real-life situations.

## Introduction

### Background

Alcohol use among adolescents is a serious public health problem in the United States [1]. Data from the High School Youth Risk Behavior Survey indicates that 22% of high school students currently drink alcohol, with more female students than male students reporting alcohol use (24% vs 20%) [2]. By grade 12, about 60% of high school students have experimented with alcohol and approximately 32% currently drink alcohol [3]. Accidents and injuries are the leading cause of death among adolescents and often related to drinking alcohol [1]. Drinking alcohol, especially often, and at an early age, negatively affects adolescents’ developing brain, learning, attention, and decision-making processes [4-6]. Adolescents who consume alcohol are also at an increased risk of physical or sexual assault and unprotected sex and, in the long term, heart disease and the early onset of certain types of cancer [7,8]. Thus, preventing and reducing adolescent alcohol use would contribute to substantial health benefits and prevent major health morbidity and mortality. With this aim, the US Department of Health and Human Services, Healthy People 2030, has set a goal to reduce drug and alcohol misuse among adolescents, including a focus on increasing the proportion of adolescents who perceive risk associated with substance abuse [9].

### Games for Health and Interactive Narrative

Technology use is ubiquitous in adolescents’ lives, and games for health interventions show signs of promise as a powerful mode for delivering public health behavior change interventions [10-14]. Games for health typically involve the use of stand-alone interactive computer apps with gameplay characteristics that are engaging, challenging, fun to play, and enhance players’ knowledge and skills useful in real life [15-17]. Games for health interventions for adolescents vary in their aims, take different forms, and use various types of technologies, including mobile phone apps, videos and documentaries, virtual reality, and video games [18].

Results from research on games for health for adolescent substance use suggest that interactive game-based interventions enhance public health education and prevention efforts [19-23]. For example, a randomized study conducted in 6th to 8th grade classrooms with 200 students used an experimental intervention with 6 new educational modules using 3 different types of games (ie, racing, arcade-style, maze) about the science of addiction [19]. Postintervention results indicated significant increases in the intervention group’s knowledge of core concepts about the science of addiction and the effects of alcohol and other drugs on the brain when compared to the control group [19]. In another study, Jander et al [21] conducted a randomized controlled trial among 34 Dutch schools. Schools were randomized to either a control condition or a web-based, computer-tailored intervention consisting of 3 sessions that depict situations related to drinking at home, drinking in a bar, and drinking at a party. The findings indicated that the intervention was effective in reducing binge drinking among adolescents 15 to 16 years. These and other studies highlight the expanding potential for games for health to increase knowledge and promote behavior change in the area of alcohol use; however, results are mixed, and further investigation of effectiveness is needed [24-27].

Recent advances in interactive learning technologies hold particular promise for designing narrative-centered games for health that effectively deliver age-appropriate and personalized behavior change interventions [26,28,29]. Interactive learning methodologies guide problem-based learning and encourage active learning and critical thinking among adolescents [30]. Health knowledge is delivered using narrative-centered dynamic virtual simulations of participants’ everyday lives, including role-playing, that can significantly increase understanding and retention of health-related knowledge [31]. These emotionally engaging games enable participants to choose strategies, make decisions, and practice skills in realistic, immersive environments [16]. By giving players agency within a virtual environment, well-designed game-based learning environments can provide experiential learning not matched by many other behavioral health interventions [32]. Yet, despite this potential, there is a dearth of prior research beyond our own that has developed interactive narrative-centered games to reduce alcohol use among adolescents.

### Opportunities to Address Adolescent Alcohol Use in Primary Care Settings

Further, because most adolescent health problems are amenable to behavioral intervention and the majority of adolescents visit a health care provider once a year, games linked to clinic-based health information technologies hold significant potential for improving health care delivery and reducing alcohol-related risky health behaviors in adolescents [21,27]. Games for health have been tested in primary care settings and have been shown to enhance training, education, motivation, engagement, and skills [33]. For example, results from one study with undergraduate medical students showed that playing the training game effectively improved knowledge about primary care screening [34]. However, games for health related to substance use prevention or reduction have primarily been implemented outside of the clinical care environment. Thus, further research is needed concerning both innovative technology-based interventions for adolescent alcohol use and the integration into primary care [35-37].

Recognizing the potential to leverage interactive narrative technologies for games for health, we are now well positioned to design and evaluate health behavior change systems that extend the reach of clinicians to realize significant impacts on behavior change for adolescent preventive health [38,39]. This paper presents data on the Interactive Narrative System for Patient-Individualized Reflective Exploration (INSPIRE), a first-of-its-kind interactive narrative game to address adolescent alcohol use, which is being designed with the goal of integrating into primary care [31].

### The INSPIRE Intervention

INSPIRE is an interactive narrative behavioral health intervention that places adolescents in the role of a high school-age character, requiring them to make narrative-based decisions regarding risk behaviors and alcohol use. INSPIRE was iteratively designed and developed by a multidisciplinary team using feedback and pilot data from over 200 adolescents who were engaged in all aspects of the multiyear development process to inform key features of INSPIRE, including the cast of characters, virtual setting, plot, scenarios about opportunities for teens to drink, and gameplay mechanics [31]. A trailer of INSPIRE can be accessed online [40].

INSPIRE was developed using the Unity game engine (Unity Technologies) and deployed as a WebGL-based app accessible through standard web browsers, requiring no installation or specialized hardware. This deployment approach supports use across a range of devices, including laptops, desktop computers, and tablets, to facilitate accessibility in clinical and educational settings. The 3D environments, character models, and animations were created by the research team’s digital artists in collaboration with the narrative design and development team. The intervention integrates a navigable 3D environment with dialogue overlays through which players make narrative choices in response to characters. Game episodes present a series of alcohol-related social situations, ranging from indirect peer pressure to direct confrontations, which players must navigate by selecting behavioral responses that influence subsequent events. INSPIRE engages adolescents in a theoretically grounded intervention for health behavior change by leveraging the dual mechanisms of interactive narrative and 3D game technologies [31].

INSPIRE’s interactive narrative storyline unfolds across 2 game episodes and includes a series of dilemma-focused scenarios that take place during a high school party hosted by the protagonist character, Max [31]. Participants help Max navigate challenging situations involving alcohol by using a “Thinking Smart” strategy developed for this study by our multidisciplinary research team extending a previous model [41]. The Thinking Smart strategy involves Max stopping to reflect on three questions when in a challenging situation: (1) “What are my goals?” which encourages players to reflect on their goals and desired results in a particular situation, (2) “What are my choices?” prompting players to recognize and weigh the various alternatives or actions available to meet their goals, and (3) “What works for me?” which requires players to assess the potential outcomes of each choice and choose the path that most closely aligns with their goals [41].

### Hypothesis, Aims, and Objectives

INSPIRE is guided by social cognitive theory (SCT) for health behavior change that posits a triadic reciprocal model of person, behavior, and environment [42]. According to the SCT framework, beliefs in personal efficacy play a pivotal role in determining behavior across diverse domains. Perceived self-efficacy refers to beliefs in one’s capabilities to organize and execute specific courses of action [43]. “Optimal functioning involves not only acquiring knowledge and skills but also the efficacy beliefs to utilize the skills well” [42]. Mastery experiences are the key way of instilling a strong sense of efficacy. In addition to acquiring knowledge through the game presenting ways of refusing alcohol (strategic knowledge) and emphasizing the risks and potential consequences of consuming alcohol (declarative knowledge) [31], adolescents who play INSPIRE can enhance their self-efficacy by mastering a series of challenging scenarios to explore alternate strategies for handling situations in the branching storyline. SCT also posits that adolescents learn vicariously from peer modeling to develop their own habits and routines, such as by observing the modeling behaviors of the intervention’s virtual characters. For example, a virtual character in INSPIRE, Hailey, declines to drink an alcoholic beverage and instead chooses to drink water. In observational learning, a single model can transmit new ways of thinking and behaving simultaneously to all adolescent participants.

The following hypotheses were tested in this study:

1. Hypothesis 1: Playing INSPIRE will lead to increases in adolescents’ self-efficacy to avoid risky alcohol use.
2. Hypothesis 2: Playing INSPIRE will lead to increases in adolescents’ knowledge about alcohol and alcohol risk.

The aim of this study is to examine the impact of playing the INSPIRE game on high school students’ knowledge and self-efficacy to reduce alcohol use. The objectives include examining changes in adolescents’ self-efficacy to engage in behaviors to avoid risky alcohol use, and changes in adolescents’ knowledge about alcohol and alcohol risk. This study will also report on participant responses from the Reflection Tool, specifically the Thinking Smart behavioral approaches that players endorsed as potentially being useful in their own personal lives.

## Methods

### INSPIRE Intervention Design

#### Design Overview

**Figure 1.**
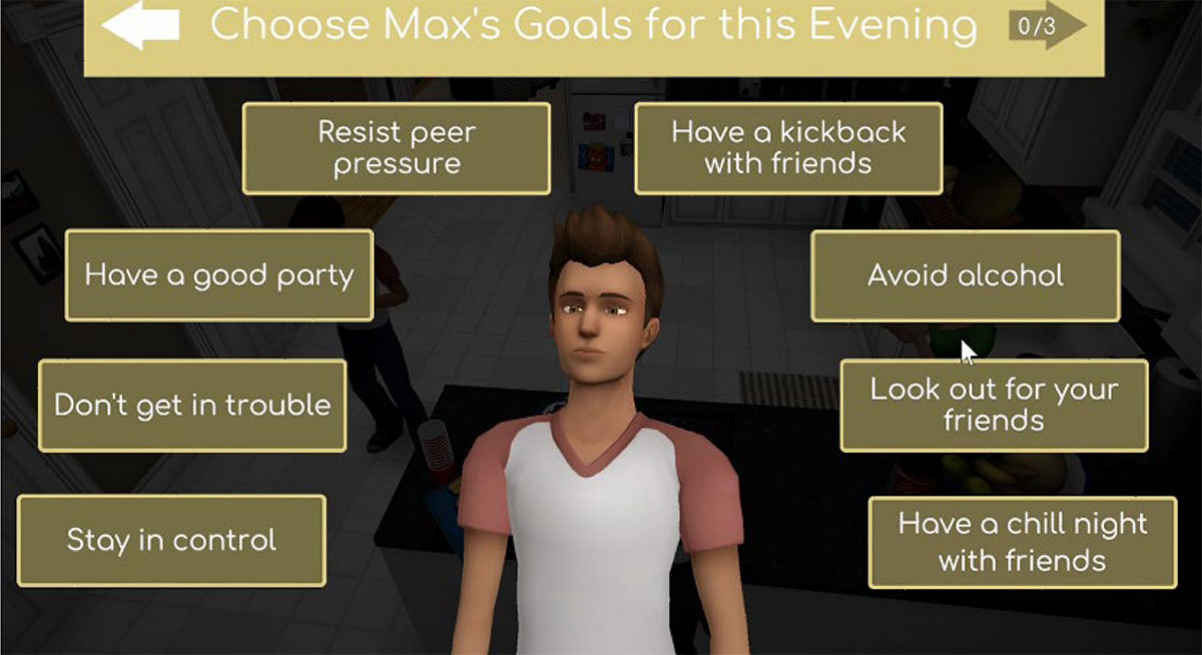
Interactive Narrative System for Patient-Individualized Reflective Exploration’s (INSPIRE) interactive page for setting up goals for the evening for Max that will help him navigate challenging situations during gameplay.

At the beginning of the INSPIRE game, as shown in Figure 1, the player sets goals for the evening for Max. During challenging interpersonal scenarios within each episode, the player is prompted 13 times in episode 1 and four times in episode 2 to make choices for Max around alcohol use and navigating peer pressure and asked to indicate whether their choices align with the goals they set for Max for the evening [31]. The choices for Max presented to the player reflect six behavioral approaches: (1) enlisting a friend for help, (2) turning down an alcoholic drink, (3) using humor to avoid consuming alcohol, (4) limiting the number of people who come over, (5) putting a different drink in your cup, and (6) suggesting a different activity. The players’ selected behavioral choices for Max influence how the interactive narrative proceeds in INSPIRE.

#### Increasing Knowledge About Alcohol Risk

**Figure 2.**
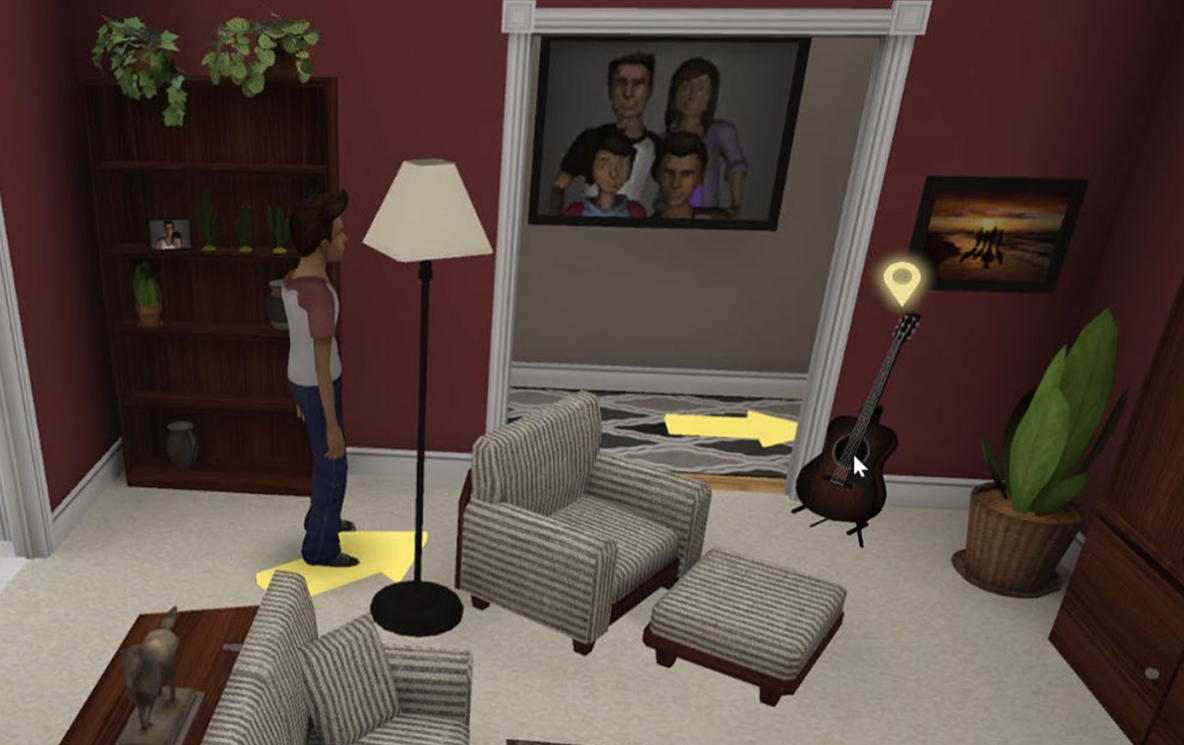
A glowing virtual object in Interactive Narrative System for Patient-Individualized Reflective Exploration’s (INSPIRE) virtual environment in Max’s home to help engage players and increase adolescents’ knowledge about alcohol-related facts.

**Figure 3.**
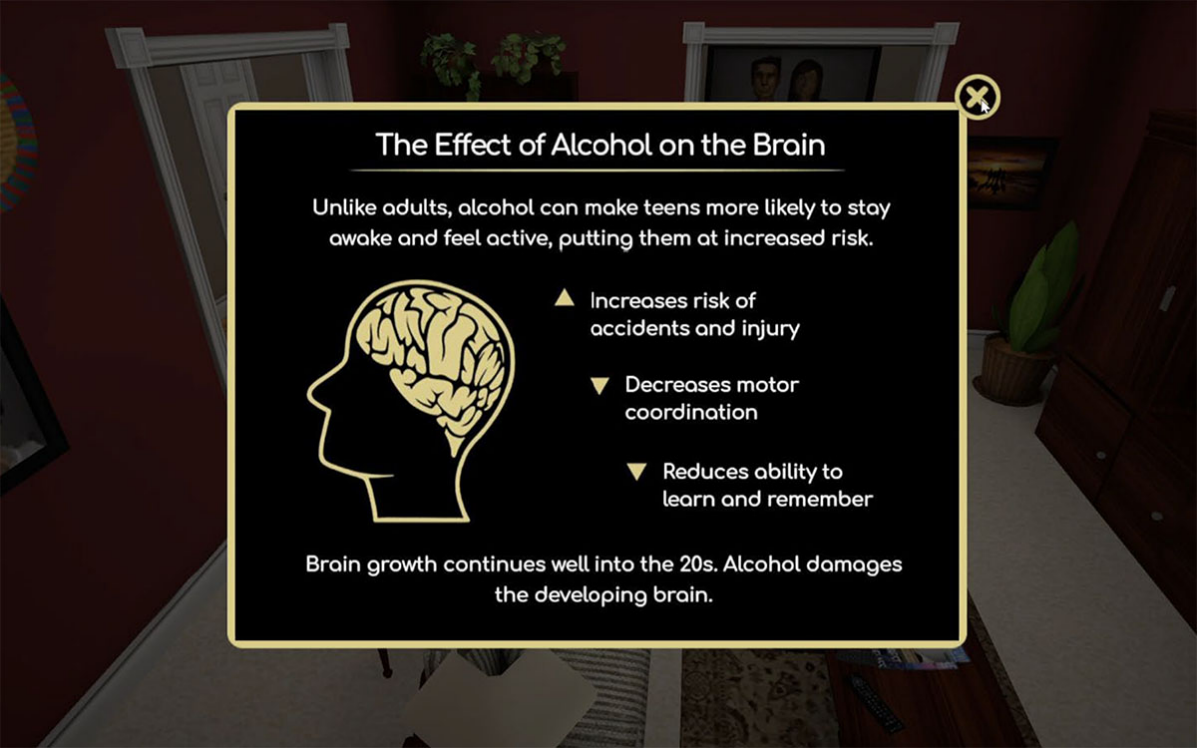
Interactive Narrative System for Patient-Individualized Reflective Exploration’s (INSPIRE) infographic to help increase adolescents’ knowledge about the effects of alcohol on the developing brain, which is revealed after clicking on the glowing virtual object.

To increase adolescents’ knowledge about alcohol, players encounter 5 key virtual objects while navigating through the 2 INSPIRE gameplay episodes. Adolescents’ interactions with these objects are optional, but visual cues (eg, a glowing marker) help to make each object salient. When the player touches the object, infographics are displayed with information about adolescent alcohol use and its consequences. For example, a glowing marker next to a package of cookies links to an infographic about the caloric content of distinct types of alcohol. As shown in Figures 2 and 3, a glowing marker next to a guitar provides access to an infographic about how alcohol affects the developing adolescent brain. Interactions with these objects also serve to foster player agency by encouraging gameplay exploration.

#### Reflecting on Alcohol Prevention Strategies

**Figure 4.**
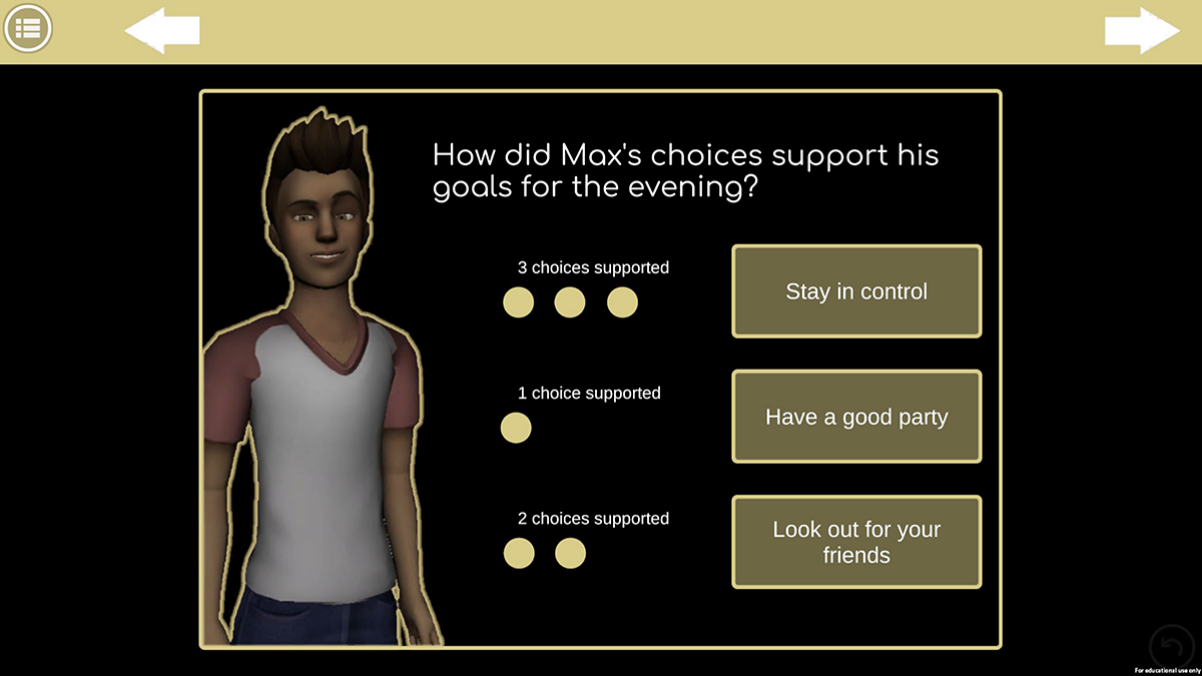
A personalized Interactive Narrative System for Patient-Individualized Reflective Exploration (INSPIRE) reflection tool review of the player’s selected choices for the protagonist, Max, and how they helped support the player’s selected goals for the evening.

**Figure 5.**
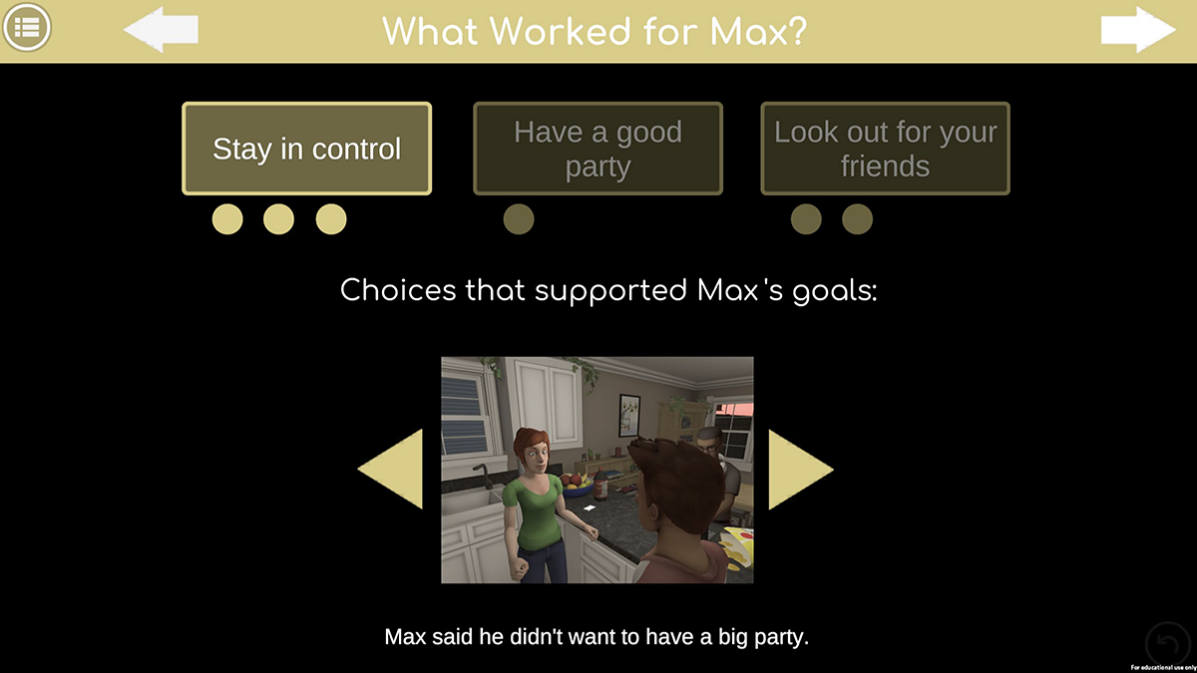
A player’s personalized Interactive Narrative System for Patient-Individualized Reflective Exploration (INSPIRE) reflection tool review of their selected behavioral choices to help reinforce strategies that can help them avoid or reduce alcohol use.

As Figures 4 and 5 show, a reflection tool appears at the end of episode 1 to help players review the strategies they used to help Max navigate challenging situations related to alcohol use [31]. Guided by the Thinking Smart behavioral approach designed for INSPIRE, adolescents are asked to reflect on their selected goals for the evening for Max and the behavioral choices made at choice points during gameplay that supported these goals. To help enhance players’ understanding and retention of these preventive strategies, the reflection tool is personalized for each player using the individual players’ gameplay and decision history for Max.

**Figure 6.**
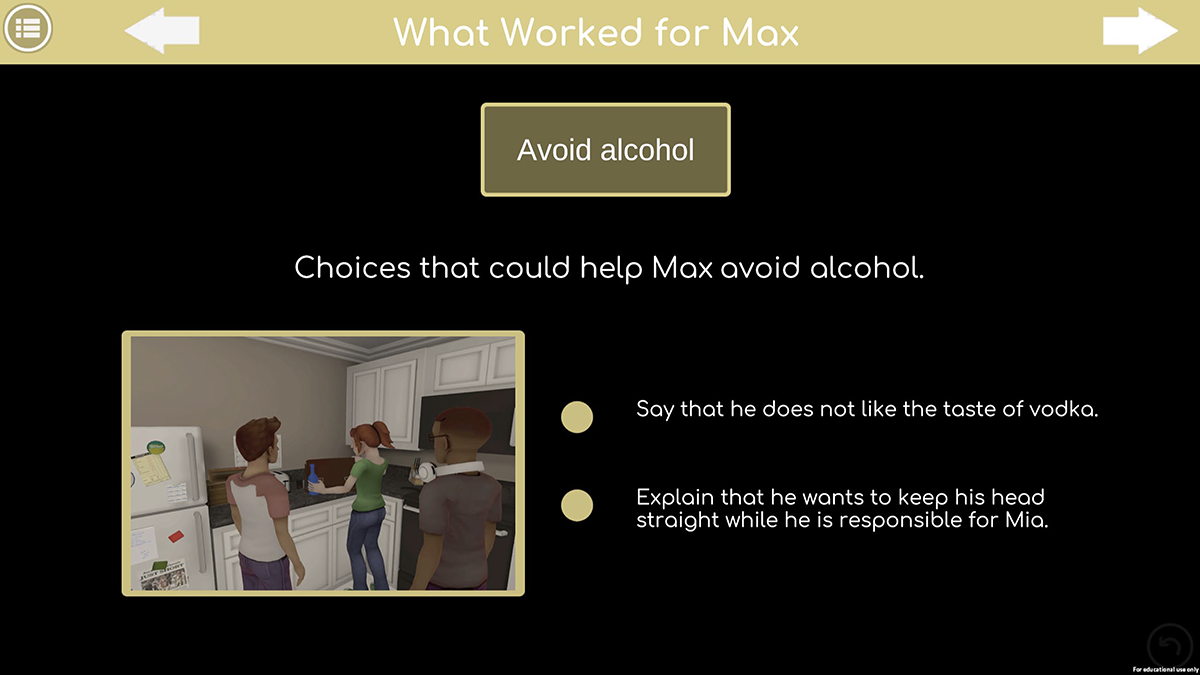
A personalized Interactive Narrative System for Patient-Individualized Reflective Exploration (INSPIRE) reflection tool review of recommended behavioral strategies for avoiding risky alcohol-related behaviors not selected by the players but selected by other players.

As Figure 6 shows, the reflection tool also generates a review of the goals and behavioral choices not selected by the player but that have been selected by other players to handle challenging situations related to Max’s alcohol use.

**Figure 7.**
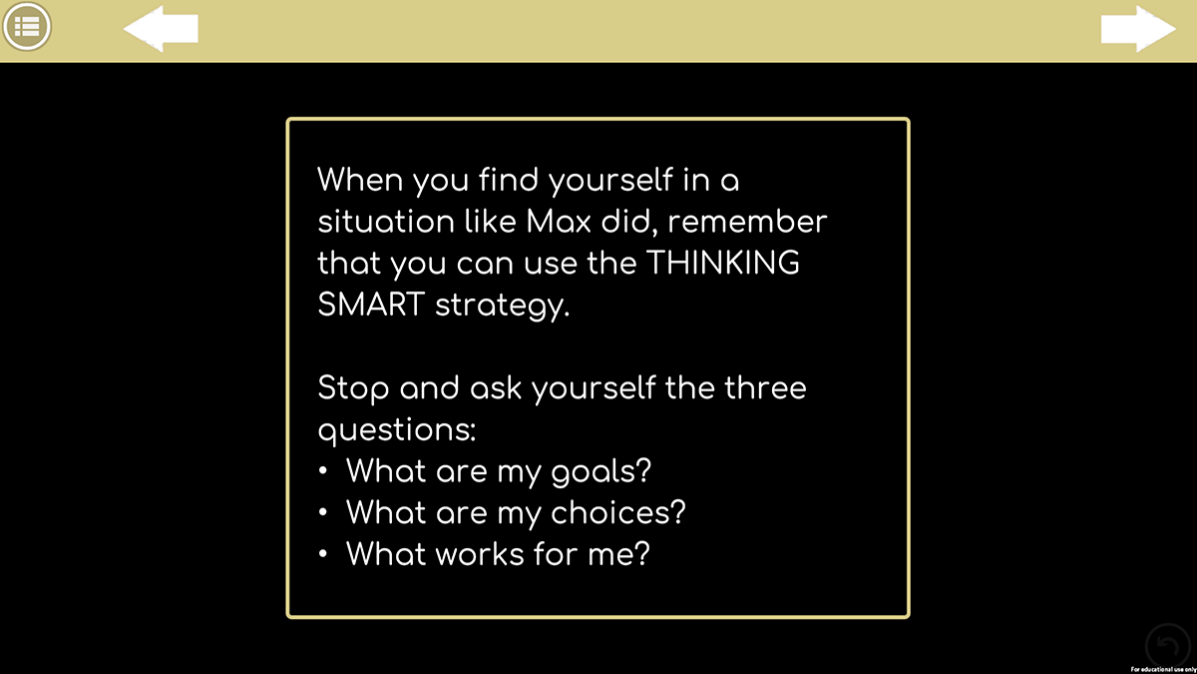
Interactive Narrative System for Patient-Individualized Reflective Exploration’s (INSPIRE) reflection tool review of the Thinking Smart strategy to help adolescents reduce risky behaviors.

Next, players are prompted to further reflect on the 6 behavioral approaches that were available to Max to help him navigate the evening. Players are asked to select the behavioral strategies that they would find useful in their own lives. In the final steps in the reflection tool, players are asked to submit free-text responses in 2 open-ended fields—“other strategies” and “thinking smart”—allowing them to contribute original ideas for alcohol resistance and harm reduction and to help enhance generalization to adolescents’ everyday lives. In 1 field, players are given the opportunity to write about any other behavioral approaches they might use that were not available in INSPIRE’s gameplay. For the second field, as shown in Figure 7, players are provided with a reminder of the 3 steps in the Thinking Smart strategy and then asked to write about when they might use Thinking Smart.

### Inclusion and Exclusion

Participants were eligible if they were aged 14 to 16 years. There were no exclusion criteria, and participation was voluntary.

### Participant Characteristics

Table 1 provides information on participants’ demographics. Participants were half female (22/44, 50%) and were aged 14 to 16 (mean 15.16, SD 0.95) years. In terms of racial and ethnic background, the largest proportion of participants identified as Hispanic or Latine (n=15, 34%), followed by White, Asian, and multiple racial or ethnic backgrounds.

**Table 1.** Means, SDs, or percentages for demographic characteristics of 44 high school students.

High school students	Values
Age, mean (SD)	15.16 (0.95)
Sex, n (%)	
Female	22 (50)
Male	22 (50)
Race or ethnicity, n (%)	
American Indian or Alaska Native	2 (4.5)
Asian or Pacific Islander	4 (9)
Black or African American	2 (4.5)
Hispanic or Latine	15 (34)
White	7 (16)
Multiple races or ethnicities	14 (32)

### Sampling Procedures

Participants were enrolled in this study using convenience sampling through an established partnership with an after-school program serving high school students in the San Francisco Bay Area. Program staff at the school distributed study information to eligible students aged 14 to 16 years. Participants were enrolled on a rolling basis.

### Ethical Considerations

#### Human Subjects Review

This study protocol was approved by the University of California San Francisco institutional review board (IRB number 19‐28893).

#### Informed Consent

Program staff obtained written informed parental consent and adolescent assent prior to participation.

#### Privacy and Confidentiality

All data were deidentified and labeled with coded study IDs, with the linkage key stored separately, and all study data were maintained on secure University of California, San Francisco (UCSF) servers with access restricted to authorized study personnel.

#### Participant Compensation

Adolescents received a US $40 gift card for their participation.

#### Participant Images

This paper does not include participants’ images.

### Sample Size, Power, and Precision

A power analysis indicated that a sample of 61 participants would be required to detect a within-subjects effect of *d*_*z*_ of 0.36 in a paired-comparison design, with *α* of .05 (2-tailed) and 80% power [44,45]. As noted in the *Participant Flow* section, intermittent data capture errors reduced available sample sizes for some secondary measures. Demographic characteristics did not differ meaningfully between the full sample and these analytic subsamples.

### Measures

#### Self-Efficacy Assessment

The INSPIRE research team, with extensive experience developing self-efficacy measures across a wide variety of domains [43,46-48], developed a 24-item self-efficacy scale to assess adolescents’ efficacy to take responsibility for their health and avoid risky alcohol use consistent with strategies in the INSPIRE game (*α*=.95). Items were piloted with adolescents in focus groups during the development phase of INSPIRE [31]. Participants rated their confidence in avoiding or resisting alcohol in a range of challenging scenarios presented in the game (eg, facing peer pressure to drink at a party). Ratings were made on a scale, with 0 indicating “not at all confident” and 10 indicating “completely confident,” and all items were combined into a composite self-efficacy score. Given the paired structure of the data and the nonnormal distribution of self-efficacy scores, the Wilcoxon signed-rank test was used to assess changes in self-efficacy from pre- to postintervention. To account for ceiling effects, 6 items with a preintervention mean ≥9 were excluded from further analysis, as such high baseline scores limit the ability to detect meaningful change. A rank-biserial correlation was also computed to estimate effect size, capturing the proportion of item-level responses that increased versus decreased after the intervention. In addition, as a component of the postsurvey, participants completed 4 supplemental self-efficacy items asking them to reflect on perceived changes in their confidence to avoid alcohol use following the intervention. Response options ranged from “Much Lower” to “Much Higher,” and the results were summarized using frequencies and percentages.

#### Knowledge Assessment

Preintervention and postintervention, participants completed 11 multiple-choice questions, derived from five virtual knowledge objects that appeared in the INSPIRE game, about the effect of alcohol use: (1) Cookies (comparing calories in alcohol), (2) Guitar (alcohol’s effects on the brain), (3) Keys (accidents and alcohol), (4) Red Solo cup (amount of alcohol in a drink cup), and (5) Vodka shot (the percentage of alcohol in drinks). These questions assessed the understanding of alcohol risks, harm-reduction strategies, and physiological effects. Responses were scored as correct (1) or incorrect (0), and overall knowledge scores were calculated by summing correct answers across items. Ten knowledge items were included in the final analysis. One question, concerning the amount of alcohol in a solo cup, was excluded due to data‐capture issues.

#### Reflection Tool and Strategy Assessment

As a component of the Reflection Tool following the completion of episode 1 gameplay, participants selected strategies that they would find useful in their own lives from a list of the 6 broad behavioral approaches in the game (ie, enlist a friend for help, limit the number of people who come over, turn down an alcoholic drink, put a different drink in your cup, use humor to avoid consuming alcohol, and suggest a different activity). The selected reflection tool strategies were transformed into a binary (0=not selected, 1=selected) using one-hot encoding. After transformation, the data were analyzed using K-means clustering.

#### Engagement Metrics

Participants completed 24 items derived from the User Engagement Scale (UES) [49,50]. These items represent 3 subscales: the Satisfaction subscale consisted of 8 items (*α*=.84; eg, I felt interested in the game, and I continued to play out of curiosity), Perceived Usability consisted of 8 items (*α*=.90; eg, I felt discouraged while playing, and the game was mentally taxing), and Immersion consisted of 7 items (*α*=0.81; eg, I lost track of time, and I was drawn into the game). Negatively worded engagement items (eg, I felt discouraged) were reverse coded. Responses ranged from 1=strongly disagree to 5=strongly agree, with higher scores reflecting greater engagement.

#### Interaction Trace Log Analysis

Digital trace logs captured participants’ interactions with the intervention, including goals and choices made, total time spent completing the game, and frequency of use of interactive elements. Descriptive analysis of these logs provided insight into how participants navigated through the intervention and engaged with key content, allowing researchers to analyze participants’ individual gameplay learning experiences as well as assess overall system interaction.

### Data Collection

Facilitated by a trained research assistant, participants completed intervention gameplay and pretest and posttest assessment measures via web access on a computer while in an after-school program classroom at their high school.

### Conditions and Design

The current study describes results from a pretest and posttest examination of the INSPIRE intervention.

### Analytic Strategy

#### Overview

From pretest to posttest, we hypothesized that playing INSPIRE would lead to (1) higher self-efficacy to avoid risky alcohol use and (2) increased knowledge about alcohol risk. Demographic information—including age, gender identity, and racial and ethnic background—was summarized using means, SDs, and frequency distributions. All statistical analyses were performed in Python (version 3.12). Data analyses were handled using the pandas and numpy (version 2.2.6) libraries, while inferential statistics were conducted using functions from the scipy.stats package. Statistical significance was set at *α*=.05 for all analyses.

#### Reflection Tool and Strategy Assessment

The Scikit-learn library in Python was used to execute K-means clustering to identify distinct patterns of strategy selection. K-means was selected for its interpretability and parsimony given the exploratory nature and modest sample size of this analysis; alternative approaches, such as k-modes or hierarchical clustering, may be examined in future work with larger samples [51,52]. Using the elbow method by plotting the total within-cluster sum of squares versus values of *k* helped determine the optimal number of clusters. A prominent elbow was observed at *k*=4, indicating that additional clusters beyond 4 provided negligible improvement in model performance. The cluster sizes were well distributed, with no group representing less than 18%. The 4-cluster solution was retained as the most interpretable grouping that preserved meaningful behavioral distinctions. Cluster distinctiveness was assessed by examining between-cluster differences in strategy endorsement patterns to confirm that each cluster reflected a qualitatively different configuration of endorsed approaches. The number of clusters illustrates meaningful behavioral patterns that could be obscured if we collapsed them into 3 clusters. Each cluster was classified by assessing members’ preferences for using direct, subtle, discreet, or more than 1 management strategy. Given the exploratory and descriptive nature of the clustering analysis, no inferential statistics are reported for cluster membership; the clusters are preliminary pattern groupings rather than definitive classifications.

## Results

### Participant Flow

**Figure 8.**
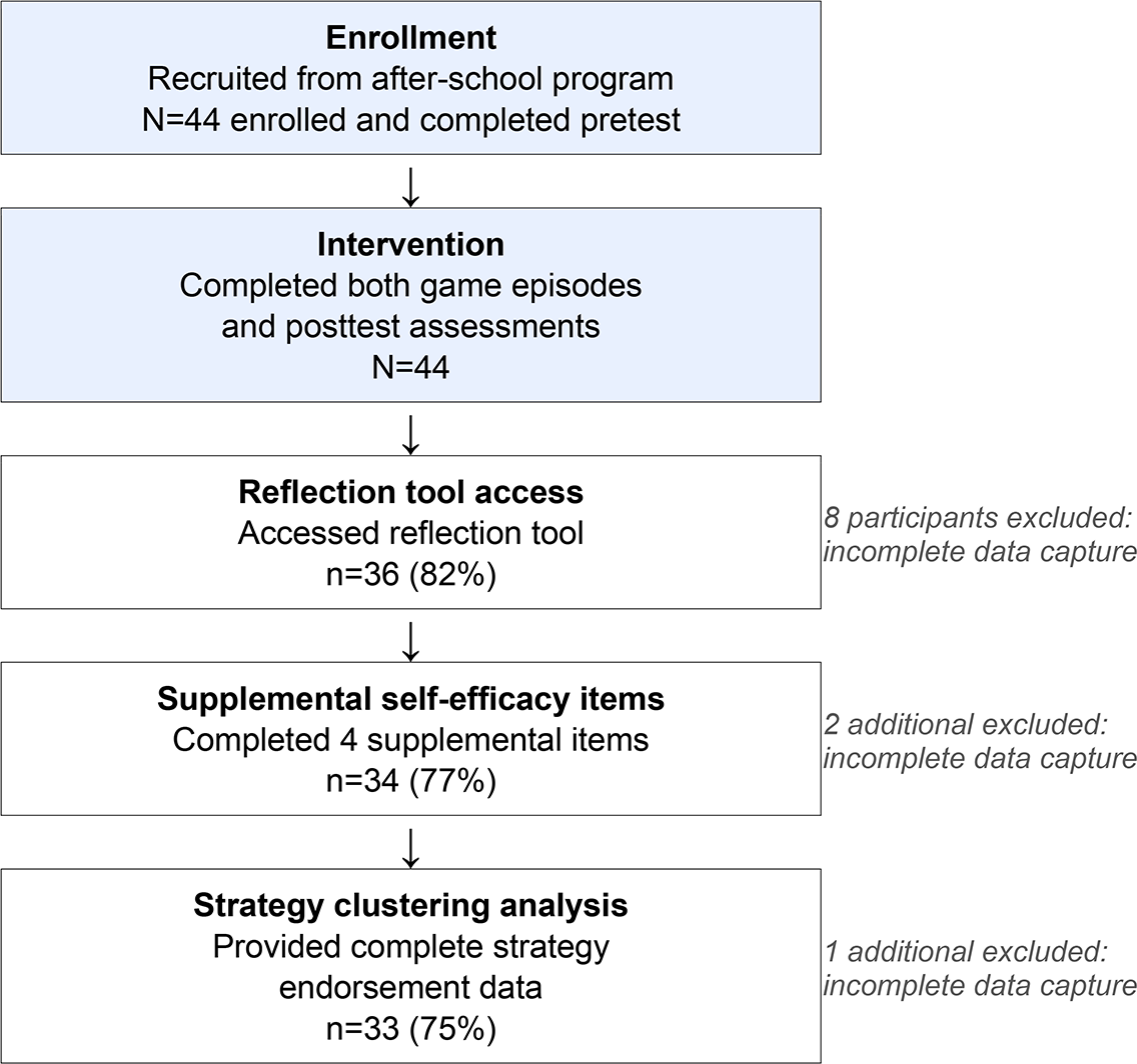
Participant flow through the Interactive Narrative System for Patient-Individualized Reflective Exploration (INSPIRE) interactive narrative alcohol prevention intervention. Single-group pre-post study with 44 high school students (aged 14-16 years), San Francisco Bay Area, 2024. Attrition at each stage resulted from intermittent data capture errors rather than participant withdrawal.

A total of 44 participants completed the pretest and posttest self-efficacy and knowledge assessments with no missing data on these primary outcomes (Figure 8). Participation data for subsequent intervention components were partially affected by intermittent game data capture errors: 34 (77%) had recorded responses on the supplemental self-efficacy items, 36 (82%) had complete Reflection Tool access logs, and 33 (75%) had usable strategy endorsement data included in the clustering analysis.

### Recruitment

Recruitment for this pre-post study occurred between April 2024 and December 2024.

### Statistics and Data Analysis

#### Analysis Overview

Missingness predominantly resulted from intermittent technical logging failures rather than participant nonresponse. Consistent with this, the Little MCAR test indicated no systematic association in the missingness pattern (*χ*²_43_=19.36; *P*>.99), supporting an assumption of data missing completely at random. Participants with incomplete data were excluded on a per-analysis basis, and no imputation was performed.

#### Alcohol Use

Regarding alcohol consumption, approximately 25% (n=11, 95% CI 14.6%-39.4%) of study participants reported having consumed alcohol at least once, and 23% (n=10, 95% CI 12.8%-37.0%) indicated alcohol use within the past year.

#### Primary Outcome: Self-Efficacy

Self-efficacy scale scores increased on average from 7.97 (SD 2.24) to 8.72 (SD 1.58) from pretest to posttest, respectively. The Wilcoxon signed-rank test (*z* score=3.436, *P*<.001, *r*=0.887) indicated that this improvement was statistically significant, with a mean difference of 0.749 (95% CI 0.590-0.909). Of the subset of teens (n=34) who completed the 4 supplemental self-efficacy items, reflecting on perceived changes in their confidence to avoid alcohol use following the intervention, the majority indicated an increased confidence following the intervention. Specifically, the majority (n=25, 74%) felt more confident in their ability to engage with friends for help, 24 (71%) felt more confident in their ability to stay under control at a party with alcohol, 21 (62%) indicated greater confidence in their ability to refuse direct alcohol offers, and 20 (59%) reported an improvement in confidence in abstaining from drinking even when others were drinking alcohol. Only 1 (3%) participant indicated a decrease in confidence across any of these scenarios.

### Primary Outcome: Knowledge

Four of the 10 items—those addressing alcohol’s caloric content, adolescent brain development, alcohol‐related accidents among teen drivers, and general alcohol effects on teens—showed significant gains in correct responses from pretest to posttest (McNemar *P*=.03, *P*=.02, *P*=.04, and *P*=.04, respectively; all confirmed by sign tests). The remaining 6 items exhibited modest numerical increases but did not reach statistical significance. Differences in the composite knowledge scores, created by summing correct responses across the 10 retained items, were significant. Scores rose from an average of 5.09 correct (median 5.0, IQR 4.0-6.0) at pretest to 6.11 correct (median 7.0, IQR 5.0-8.0) at posttest, a mean change of 1.02 (SD 2.35; 95% CI 0.31-1.74). This improvement was confirmed by the Wilcoxon signed‐rank test (*z* score=3.804, *P*<.001, *r*=0.567) and a sign test on the composite (*P*<.001). Trace log data elucidated that participants engaged with the embedded knowledge objects, with 82% (n=36, 95% CI 68.0%-90.5%) of participants interacting with all available objects.

### Reflection Tool

Of the 44 study participants, 33 (75%) provided complete and usable strategy endorsement data through the reflection tool; 11 participants were excluded from the clustering analysis due to incomplete reflections or missing log entries. Application of K-means clustering (*k*=4) to these participants’ strategy profiles yielded 4 groups, each characterized by a distinct pattern of endorsed behavioral approaches (Table 2). Within the first of 4 clusters, members used more *direct strategies* to help Max avoid alcohol use, which included limiting the number of party guests or asking a friend for help with challenging alcohol-related scenarios. Adolescents in cluster 2 relied on *subtle social tactics* such as using humor to avoid alcohol use or guiding the group toward activities that did not involve drinking. Group 3 teens favored *discreet methods*, such as switching to nonalcoholic beverages. The fourth group *integrated both direct and subtle strategies* and adjusted their approach according to the context.

**Table 2.** Exploratory cluster characteristics from K-means analysis of reflection tool strategy endorsement profiles from 33 adolescent participants.

Cluster	Participants, n (%)	Predominant approach	Key strategies endorsed
1	11 (33)	Direct refusal and boundary-setting	Limiting guests, turning down drinks, suggesting alternatives, enlisting a friend
2	8 (24)	Subtle social redirection	Using humor, suggesting alternative activities, limiting guests
3	8 (24)	Discreet substitution and environmental management	Substituting a nonalcoholic beverage, turning down drinks, limiting guests
4	6 (18)	Integrated direct and subtle strategies	Moderate endorsement across multiple strategies without strong reliance on any single approach

### User Engagement

Participants spent an average of 52.7 (SD 13.5) minutes completing both gameplay episodes. (While episode-specific time data were not collected for this sample, previous playtesting indicates that episode 1 takes approximately 30 minutes to complete, and episode 2 takes approximately 20 minutes.) The User Satisfaction subscale scores averaged 3.97 (SD 0.52, 95% CI 3.81-4.13), and Perceived Usability scores averaged 3.19 (SD 0.92, 95% CI 2.91-3.47). The Immersion subscale yielded a mean of 3.21 (SD 0.72, 95% CI 2.99, 3.43).

## Discussion

### Principal Findings

This study examined the effects of INSPIRE, a first of its kind interactive narrative-centered intervention to reduce adolescent alcohol use, on adolescent self-efficacy and knowledge about alcohol risk. Pre-post study analysis supported our hypotheses suggesting that by setting goals, exploring alternative behavioral strategies, and virtually experiencing alcohol-related situations with peer pressure, adolescents who played INSPIRE (1) enhanced their self-efficacy beliefs in avoiding risky alcohol use and (2) increased their knowledge about alcohol risk.

An analysis of adolescents’ use of the Reflection Tool to help players reinforce strategies presented in the INSPIRE game and generalize the strategies to their own lives found that adolescents gravitated toward different behavioral approaches when faced with choices about alcohol use. These included switching between direct, for example, asking a friend for help, and subtle strategies, for example, using humor, depending on the context. Consistent with our previous findings [29,31], the study also found that adolescents were engaged in the INSPIRE gameplay. In general, these findings suggest that interactive narrative interventions can be an effective approach to mitigating alcohol risk behaviors among adolescents [28].

As elucidated in social cognitive theory, beyond enhancing knowledge, personal self-efficacy to navigate challenging situations, is predictive of actual behavior managing potential risks related to alcohol use [31]. Building on recent game-based and survey research emphasizing the association between self-efficacy and reductions in drinking, our findings underscore the role that digital health interventions, using interactive narratives such as INSPIRE, can play in strengthening self-efficacy among teens [24,26,53].

Previous interventions have aimed to increase adolescents’ self-efficacy in refusing alcohol through conventional approaches, such as skills training, role-playing, or brief computer-based simulations [54,55]. The use of digital interactive narratives, especially those that enable adolescents to engage with a dynamic storyline that responds to their choices, is a technology that can improve teen engagement but is underexplored in the health literature [56]. Interactive computer narratives offer a novel approach to enhance prevention through primary care [39].

The findings from the reflection tool indicating that a total of 4 clusters represented unique approaches to managing risky scenarios related to alcohol use, ranging from direct refusal and boundary-setting to subtle social redirection to discreet behavioral substitution, have the potential to inform personalized tailored intervention content to contribute to engagement and relevance to their lives [57]. Although the modest analytic sample (n=33) and exploratory design preclude definitive classification, these preliminary patterns are consistent with the view that adolescents bring different strategic orientations to peer-pressure contexts rather than uniformly adopting a single approach [58]. Further research examining these strategy patterns with larger samples is ongoing and should clarify whether these configurations are stable across populations and whether they relate to longitudinal engagement, self-efficacy, or behavioral outcomes.

The high engagement in the interactive gameplay of INSPIRE reflects the co-design of the interactive narrative plots, scenes, characters, and dialogue with adolescents from a wide range of backgrounds [59]. While most participants reported positive experiences, a small portion noted occasional difficulty—such as feeling unable to complete certain tasks. These results suggest that, overall, the intervention was well received, though certain areas may benefit from refinement to improve ease of use and deepen engagement [60].

While this study indicates that INSPIRE has the potential to enhance self-efficacy and knowledge, future studies are needed to assess actual skill or behavior change. In addition, while the results indicate significant increases in self-efficacy from preintervention to postintervention, 33 (75%) of the adolescent participants reported never drinking. Having friends who do not consume alcohol is a protective factor and may have influenced students’ relatively high rates of self-efficacy to navigate alcohol-related scenarios pre-INSPIRE gameplay and suggests that a wider range of adolescent respondents and alcohol use experience would be useful for future studies [61].

### Generalizability

A related but broader limitation is that teen recruitment from a single school or after-school program may limit the generalizability of results [62]. In addition, the achieved sample of 44 fell below the 61 participants the power analysis indicated were needed to detect a within-subjects effect of *d_z_* of 0.36 at 80% power [44]. The shortfall stemmed from lower-than-anticipated consent rates and from technological barriers during data collection, including problems with access ID distribution that prevented some adolescents from participating. The observed pre-post effects on the self-efficacy and knowledge item composites were nonetheless large enough to reach statistical significance, indicating the achieved sample retained sensitivity to detect changes of the observed magnitude [45]. The reduced sample, however, limits the precision of effect size estimates and may have constrained detection of smaller item-level changes [45]. Finally, this study was conducted in a high school after-school setting to conduct a preliminary pre-post study examination of the effects of the INSPIRE game. However, as INSPIRE is designed to be ultimately integrated into primary care, future examinations of INSPIRE will build on this study to involve adolescents completing the intervention at home after visiting their primary care provider.

### Conclusions

As a first of its kind interactive narrative intervention, INSPIRE offers an innovative theoretically grounded model for supporting adolescent health behavior change. This study, providing preliminary findings on the effects of INSPIRE, enhances our understanding of how to use innovative learning technologies to help reduce risky alcohol use. INSPIRE enables adolescents to actively explore and practice responses to realistic social situations through a branching narrative experience grounded in social cognitive theory. Extending prior research in the field through using personalized narrative adaptations, this study indicates that through reinforcing goals and decisions to avoid risky behavior, adolescents can enhance their self-efficacy beliefs to avoid risky alcohol use and increase their knowledge about alcohol risk.

Implications of these study outcomes, including findings from the “Reflection Tool” indicating that adolescents gravitate toward different behavioral strategies depending on the context, include the potential to facilitate the generalization of preventive behaviors to real-life situations [38]. Further, implications for primary care practice include leveraging adolescent well-visits to prescribe evidence-based interventions such as INSPIRE to assess and intervene in risky drinking and enhance prevention efforts [63]. Important next steps in the development of INSPIRE include the implementation of the game into adolescent primary clinics, as well as an evaluation of the behavioral effects of INSPIRE on adolescent alcohol use.

## Abbreviations

INSPIRE: Interactive Narrative System for Patient-Individualized Reflective Exploration
SCT: social cognitive theory
UCSF: University of California, San Francisco
UES: User Engagement Scale

## References

[1] (2025). About underage drinking. Centers for Disease Control and Prevention (CDC).

[2] (2024). Youth risk behavior surveillance system data summary & trends report: 2013-2023. https://www.cdc.gov/yrbs/dstr/index.html.

[3] (2021). Youth online: high school YRBS. Centers for Disease Control and Prevention (CDC).

[4] Lees B, Meredith LR, Kirkland AE, Bryant BE, Squeglia LM (2020). Effect of alcohol use on the adolescent brain and behavior. Pharmacol Biochem Behav.

[5] Spear LP (2018). Effects of adolescent alcohol consumption on the brain and behaviour. Nat Rev Neurosci.

[6] White A, Hingson R, Sloboda Z, Petras H, Robertson E, Hingson R (2019). Prevention of Substance Use.

[7] Waterman EA, Lee KDM, Edwards KM (2019). Longitudinal associations of binge drinking with interpersonal violence among adolescents. J Youth Adolescence.

[8] Shiels MS, Haque AT, Berrington de González A (2025). Trends in cancer incidence and mortality rates in early-onset and older-onset age groups in the United States, 2010-2019. Cancer Discov.

[9] Increase the proportion of adolescents who think substance abuse is risky—SU-R01. Healthy People 2030.

[10] Haruna H, Hu X, Chu SKW, Mellecker RR, Gabriel G, Ndekao PS (2018). Improving sexual health education programs for adolescent students through game-based learning and gamification. Int J Environ Res Public Health.

[11] Lehtimaki S, Martic J, Wahl B, Foster KT, Schwalbe N (2021). Evidence on digital mental health interventions for adolescents and young people: systematic overview. JMIR Ment Health.

[12] Rose T, Barker M, Maria Jacob C (2017). A systematic review of digital interventions for improving the diet and physical activity behaviors of adolescents. J Adolesc Health.

[13] Westley M, Laffier J, Rehman A, Eriksen G, Sønderby R, Sørdahl M (2026). Designing evidence-based digital mental health check-ins in higher education. Acad Ment Health Well-Being.

[14] Choi J, Tamí-Maury I, Cuccaro P, Kim S, Markham C (2023). Digital health interventions to improve adolescent HPV vaccination: a systematic review. Vaccines (Basel).

[15] Wattanasoontorn V, Boada I, García R, Sbert M (2013). Serious games for health. Entertain Comput.

[16] Warsinsky S, Schmidt-Kraepelin M, Rank S, Thiebes S, Sunyaev A (2021). Conceptual ambiguity surrounding gamification and serious games in health care: literature review and development of game-based intervention reporting guidelines (GAMING). J Med Internet Res.

[17] Jiang C, Dietrich T, Durl J, Saleme P, Hrynyschyn R, Stock C (2026). Virtual reality in adolescent alcohol and other drugs education programs: a systematic scoping review. J Adolesc Health.

[18] Ozer EM (2026). The potential and challenges of interactive virtual environments to support behavior change in adolescents. J Adolesc Health.

[19] Epstein J, Noel J, Finnegan M, Watkins K (2016). Bacon brains: video games for teaching the science of addiction. J Child Adolesc Subst Abuse.

[20] Sanchez RP, Bartel CM (2015). The feasibility and acceptability of “Arise”: an online substance abuse relapse prevention program. Games Health J.

[21] Jander A, Crutzen R, Mercken L, Candel M, de Vries H (2016). Effects of a web-based computer-tailored game to reduce binge drinking among Dutch adolescents: a cluster randomized controlled trial. J Med Internet Res.

[22] Doumas DM, Esp S, Turrisi R, Bond L, Glenn SD (2025). A randomized controlled trial testing the efficacy of the eCHECKUP TO GO on drinking games participation and behavior among high school seniors. Addict Behav.

[23] Ho FK, Tung KTS, Wong RS (2021). An internet quiz game intervention for adolescent alcohol drinking: a clustered RCT. Pediatrics.

[24] Engelbrecht H, van der Laan LN, van Enschot R, Krahmer E (2022). The role of agency and threat immediacy in interactive digital narrative fear appeals for the prevention of excessive alcohol use: randomized controlled trial. JMIR Serious Games.

[25] Martínez-Miranda J, Espinosa-Curiel IE (2022). Serious games supporting the prevention and treatment of alcohol and drug consumption in youth: scoping review. JMIR Serious Games.

[26] Yap AGH, Roy RED, Lasala JRS (2020). Evaluation of a cognitive-behavioral game design-based mobile game on alcohol use for adolescents. Games Health J.

[27] Lee S, Kim J, Bockhold S, Lee J, Chun J, Yu M (2024). Game-based digital interventions for enhancing positive development and addressing substance use in adolescents: a systematic review. Children (Basel).

[28] Sinha A, Rowe J, Ozer E (2024). Narrative play—promoting adolescent health with interactive games. JAMA.

[29] Pugatch M, Blum NJ, Barbaresi WJ (2023). Engagement of adolescents with ADHD in a narrative-centered game-based behavior change environment to reduce alcohol use. Front Educ.

[30] Mancone S, Corrado S, Tosti B, Spica G, Diotaiuti P (2024). Integrating digital and interactive approaches in adolescent health literacy: a comprehensive review. Front Public Health.

[31] Ozer EM, Rowe J, Tebb KP (2020). Fostering engagement in health behavior change: iterative development of an interactive narrative environment to enhance adolescent preventive health services. J Adolesc Health.

[32] Dever D, Wiedbusch M, Marano C (2024). From product to process data: game mechanics for science learning. Int J Serious Games.

[33] Sharifzadeh N, Kharrazi H, Nazari E (2020). Health education serious games targeting health care providers, patients, and public health users: scoping review. JMIR Serious Games.

[34] Tubelo RA, Portella FF, Gelain MA (2019). Serious game is an effective learning method for primary health care education of medical students: a randomized controlled trial. Int J Med Inform.

[35] Jordan HR, Sahni S, Ahmed MM (2023). A comprehensive literature review of digital health interventions in the treatment of substance use disorder with special focus on mobile applications. Cureus.

[36] Ramsey AT, Satterfield JM, Gerke DR, Proctor EK (2019). Technology-based alcohol interventions in primary care: systematic review. J Med Internet Res.

[37] Lee AK, Bobb JF, Richards JE (2023). Integrating alcohol-related prevention and treatment into primary care: a cluster randomized implementation trial. JAMA Intern Med.

[38] Rowe JP, Lester JC (2020). Artificial intelligence for personalized preventive adolescent healthcare. J Adolesc Health.

[39] Wong CA, Madanay F, Ozer EM (2020). Digital health technology to enhance adolescent and young adult clinical preventive services: affordances and challenges. J Adolesc Health.

[40] (2026). INSPIRE trailer. The IntelliMedia Group YouTube page.

[41] Ozer MN (1982). The Ozer Method: A Breakthrough Problem-Solving Technique for Parents and Children.

[42] Bandura A (1997). Self-Efficacy: The Exercise of Control.

[43] Ozer EM, Bandura A (1990). Mechanisms governing empowerment effects: a self-efficacy analysis. J Pers Soc Psychol.

[44] Faul F, Erdfelder E, Lang AG, Buchner A (2007). G*Power 3: a flexible statistical power analysis program for the social, behavioral, and biomedical sciences. Behav Res Methods.

[45] Cohen J (2013). Statistical Power Analysis for the Behavioral Sciences.

[46] Ozer EM (1995). The impact of childcare responsibility and self-efficacy on the psychological health of professional working mothers. Psychol Women Q.

[47] Ozer EM, Adams SH, Gardner LR, Mailloux DE, Wibbelsman CJ, Irwin CE (2004). Provider self-efficacy and the screening of adolescents for risky health behaviors. J Adolesc Health.

[48] Ozer EM, Adams SH, Orrell-Valente JK (2011). Does delivering preventive services in primary care reduce adolescent risky behavior?. J Adolesc Health.

[49] O’Brien HL, Cairns P, Hall M (2018). A practical approach to measuring user engagement with the refined User Engagement Scale (UES) and new UES short form. Int J Hum Comput Stud.

[50] Wiebe EN, Lamb A, Hardy M, Sharek D (2014). Measuring engagement in video game-based environments: investigation of the User Engagement Scale. Comput Human Behav.

[51] Suyal M, Sharma S (2024). A review on analysis of k-means clustering machine learning algorithm based on unsupervised learning. J Artif Intell Syst.

[52] Haraty RA, Dimishkieh M, Masud M (2015). An enhanced k-means clustering algorithm for pattern discovery in healthcare data. Int J Distrib Sens Netw.

[53] De Jans S, Hudders L, Van de Sompel D (2026). Adolescents’ intention to reduce alcohol use: a health belief model approach to cancer prevention. Alcohol Alcohol.

[54] Schwinn TM, Schinke SP, Keller B, Hopkins J (2019). Two- and three-year follow-up from a gender-specific, web-based drug abuse prevention program for adolescent girls. Addict Behav.

[55] Guldager JD, Kjær SL, Grittner U, Stock C (2022). Efficacy of the virtual reality intervention VR FestLab on alcohol refusal self-efficacy: a cluster-randomized controlled trial. Int J Environ Res Public Health.

[56] Nicholas J, Mills B, Hansen S (2022). Developing an alcohol and other drug serious game for adolescents: considerations for improving student engagement. Aust N Z J Public Health.

[57] Haney K, Borger T, Bursac V (2025). Identifying adaptations to an mHealth alcohol reduction intervention for reducing alcohol use in adolescent and young adult cancer survivors: qualitative study. JMIR Cancer.

[58] Trucco EM, Hartmann SA, Fallah-Sohy N (2024). Charting a course for empowered adolescent substance use treatment. Clin Psychol (New York).

[59] The Lancet Digital Health (2023). Children must co-design digital health research. Lancet Digit Health.

[60] O’Brien HL, Toms EG (2010). The development and evaluation of a survey to measure user engagement. J Am Soc Inf Sci.

[61] Norman I, Gloppen K (2025). The role of peers in alcohol use among youth in Minnesota. https://www.health.state.mn.us/communities/alcohol/documents/alcoholpeers.pdf.

[62] Linton JD (2020). The limits of generalizability: why good theory can have bad outcomes. Technovation.

[63] Calihan JB, Bagley SM (2026). Pediatrics for Underserved Populations.

